# Lightweight Transfer Learning Models for Multi-Class Brain Tumor Classification: Glioma, Meningioma, Pituitary Tumors, and No Tumor MRI Screening

**DOI:** 10.1007/s10278-025-01686-1

**Published:** 2025-09-19

**Authors:** Alon Gorenshtein, Tom Liba, Avner Goren

**Affiliations:** 1 Azrieli Faculty of Medicine, Bar-Ilan University, Safed, 1311502 Israel; 2 Rambam Health Care Campus, Haifa, Israel; 3 Maccabi Healthcare Services, Tel Aviv, Israel

**Keywords:** Deep learning, Brain tumor, Glioma, Meningioma, Pituitary tumor, Artificial intelligence

## Abstract

Glioma, pituitary tumors, and meningiomas constitute the major types of primary brain tumors. The challenge in achieving a definitive diagnosis stem from the brain’s complex structure, limited accessibility for precise imaging, and the resemblance between different types of tumors. An alternative and promising solution is the application of artificial intelligence (AI), specifically through deep learning models. We developed multiple lightweight deep learning models ResNet-18 (both pretrained on ImageNet and trained from scratch), ResNet-34, ResNet-50, and a custom CNN to classify glioma, meningioma, pituitary tumor, and no tumor MRI scans. A dataset of 7023 images was employed, split into 5712 for training and 1311 for validation. Each model was evaluated via accuracy, area under the curve (AUC), sensitivity, specificity, and confusion matrices. We compared our models to SOTA methods such as SAlexNet and TumorGANet, highlighting computational efficiency and classification performance. ResNet pretrained achieved 98.5–99.2% accuracy and near-perfect validation metrics, with an overall AUC of 1.0 and average sensitivity and specificity both exceeding 97% across the four classes. In comparison, ResNet-18 trained from scratch and the custom CNN achieved 91.99% and 87.03% accuracy, respectively, with AUCs ranging from 0.94 to 1.00. Error analysis revealed moderate misclassification of meningiomas as gliomas in non-pretrained models. Learning rate optimization facilitated stable convergence, and loss metrics indicated effective generalization with minimal overfitting. Our findings confirm that a moderately sized, transfer-learned network (ResNet-18) can deliver high diagnostic accuracy and robust performance for four-class brain tumor classification. This approach aligns with the goal of providing efficient, accurate, and easily deployable AI solutions, particularly for smaller clinical centers with limited computational resources. Future studies should incorporate multi-sequence MRI and extended patient cohorts to further validate these promising results.

**Supplementary Information:**

The online version contains supplementary material available at 10.1007/s10278-025-01686-1.

## Introduction

Glioma, meningiomas, and pituitary tumors constitute the major types of primary brain tumors, accounting for 24.5%, 39%, and 17.1%, respectively [1]. Magnetic resonance imaging (MRI) with gadolinium enhancement is the preferred diagnostic tool for these conditions [2], with standard imaging protocols—T1-weighted, T2-weighted, T2-weighted fluid-attenuated inversion recovery (FLAIR), and post-contrast T1-weighted sequences—demonstrating good diagnostic accuracy [3]. While pituitary tumors and meningiomas typically present benign characteristics, gliomas often manifest more aggressively and account for 75% of malignant primary brain tumors in adults, with only 1% classified as non-malignant [1]. This underscores the critical need to differentiate gliomas from benign tumor types.

Despite the utility of MRI technology, definitive diagnosis remains challenging due to the brain’s complex anatomy, restricted access for precise imaging [4], and overlapping tumor characteristics. Sophisticated imaging techniques—such as diffusion-weighted MRI, perfusion-weighted MRI, magnetic resonance spectroscopy, and positron emission tomography—have been investigated to address these diagnostic challenges [5]. However, their incorporation into routine clinical practice remains limited, given the specialized expertise and infrastructure required for their interpretation [5].

Automated brain tumor classification using deep convolutional neural networks (CNNs) has emerged as a powerful approach, as these models learn features directly from imaging data without necessitating hand-crafted feature extraction [7]. Transfer learning, wherein a model is initially trained on a large-scale dataset (e.g., ImageNet) before fine-tuning on the target medical domain, has proven highly effective in various clinical imaging tasks [8–10]. Chaudhary et al. [11] introduced SAlexNet with residual attention blocks, achieving multi-class classification accuracies above 98%. Another related strategy leverages generative adversarial networks (GANs) for data augmentation: Nag et al. [12] combined ResNet-50-based feature extraction with a GAN-driven augmentation scheme (“TumorGANet”), exceeding 99% accuracy in four-class brain tumor classification. Concurrently, Qureshi et al. [13] developed an “ultra-light” CNN that integrates gray-level co-occurrence matrix (GLCM) textural descriptors, recording a ~ 99% detection rate. Haque et al. [14] employed an inverted pyramid pooling module in their NeuroNet19 architecture, obtaining 99.3% accuracy. Although these sophisticated models demonstrate remarkable performance, their higher computational demands can limit their applicability in real-time clinical settings with constrained resources.

An alternative and promising avenue lies in developing lightweight, efficient deep learning architectures capable of distinguishing between tumorous and non-tumorous MRI images [6]. Residual networks (ResNet variants) have shown particular promise due to skip connections that mitigate the vanishing gradient problem [14], and they have proven effective in tasks like medical segmentation. Nevertheless, the synergy between moderate-depth architectures (e.g., ResNet-18) and systematic data preprocessing/augmentation for multi-class brain tumor classification remains underexplored. While prior studies have focused on complex, resource-intensive models, [12, 14–16] there remains a need for efficient solutions that balance high accuracy with low computational overhead, particularly to support deployment in resource-limited clinical environments.

Hence, the overarching goal of this study is to develop and evaluate multiple lightweight and efficient deep learning models that classify MRI scans into glioma, meningioma, pituitary tumor, or non-tumorous categories. This work introduces novel adaptations of moderate-depth ResNet architectures combined with optimized preprocessing and augmentation strategies, setting it apart by prioritizing computational efficiency without compromising diagnostic precision. By providing precise, automated diagnoses, these approaches have the potential to transform neuro-oncological care, particularly in smaller clinical centers with limited computational infrastructure.

The key contributions of this study are as follows:

- Development of lightweight deep learning models based on moderate-depth ResNet variants, fine-tuned for multi-class brain tumor classification, achieving high accuracy with reduced computational requirements.
- Integration of systematic data preprocessing and augmentation techniques to enhance model robustness and generalization, addressing limitations in existing datasets.
- Comprehensive evaluation of the proposed models on benchmark MRI datasets, demonstrating superior efficiency compared to resource-heavy state-of-the-art approaches.
- Provision of an open-source framework to facilitate adoption in clinical settings, promoting accessibility for resource-constrained environments.

## Materials and Methods

### Dataset

This study developed a brain tumor detection model using a comprehensive dataset of 7,023 MRI images sourced from public dataset [17]. The dataset, compiled by Nickparvar by merging three smaller datasets, included 1,621 images for glioma, 1,645 for meningioma, 1,757 for pituitary tumors, and 2,000 for non-tumor MRI images. The goal was to train a model to recognize specific patterns indicative of tumors, thus enabling automated and accurate detection. The dataset was curated to reflect a diverse range of tumors in terms of grade, type, and presentation, enhancing the model's applicability across different clinical settings. The images, obtained from various imaging devices, contribute to the model’s generalizability.

### Data Characteristics

The MRI images used in this study varied in both orientation (horizontal, sagittal) and type (T1, T2), encompassing a wide range of anatomical perspectives and magnetic properties. This variability ensures the model is not biased towards any specific orientation or imaging parameter, enhancing its generalizability across different clinical settings and machine configurations.

### Deep Learning Approach

In this study, we employed deep learning due to its demonstrated efficacy in medical image analysis, particularly for recognizing complex patterns in imaging data. We selected a convolutional neural network (CNN), a type of deep learning model particularly adept at processing images. CNNs automate the feature extraction process, reducing the need for manual intervention and allowing for more robust data analysis.

### Preprocessing

All MRI images underwent a carefully designed preprocessing and augmentation pipeline to bolster model robustness. First, each image was resized to 256 × 256 pixels and center-cropped to 224 × 224 to maintain aspect ratio consistency. Data augmentation was then applied, which included random horizontal and vertical flips (*p* = 0.5), random rotations (± 15°), color jitter (brightness, contrast, saturation, hue), and random affine transformations (translation, scaling). Finally, all images were normalized using the standard mean and standard deviation from the ImageNet dataset (mean = [0.485, 0.456, 0.406], std = [0.229, 0.224, 0.225]). This comprehensive approach aimed to increase generalizability by exposing the model to diverse spatial and intensity variations.

### Model Selection and Implementation

**Fig. 1:**
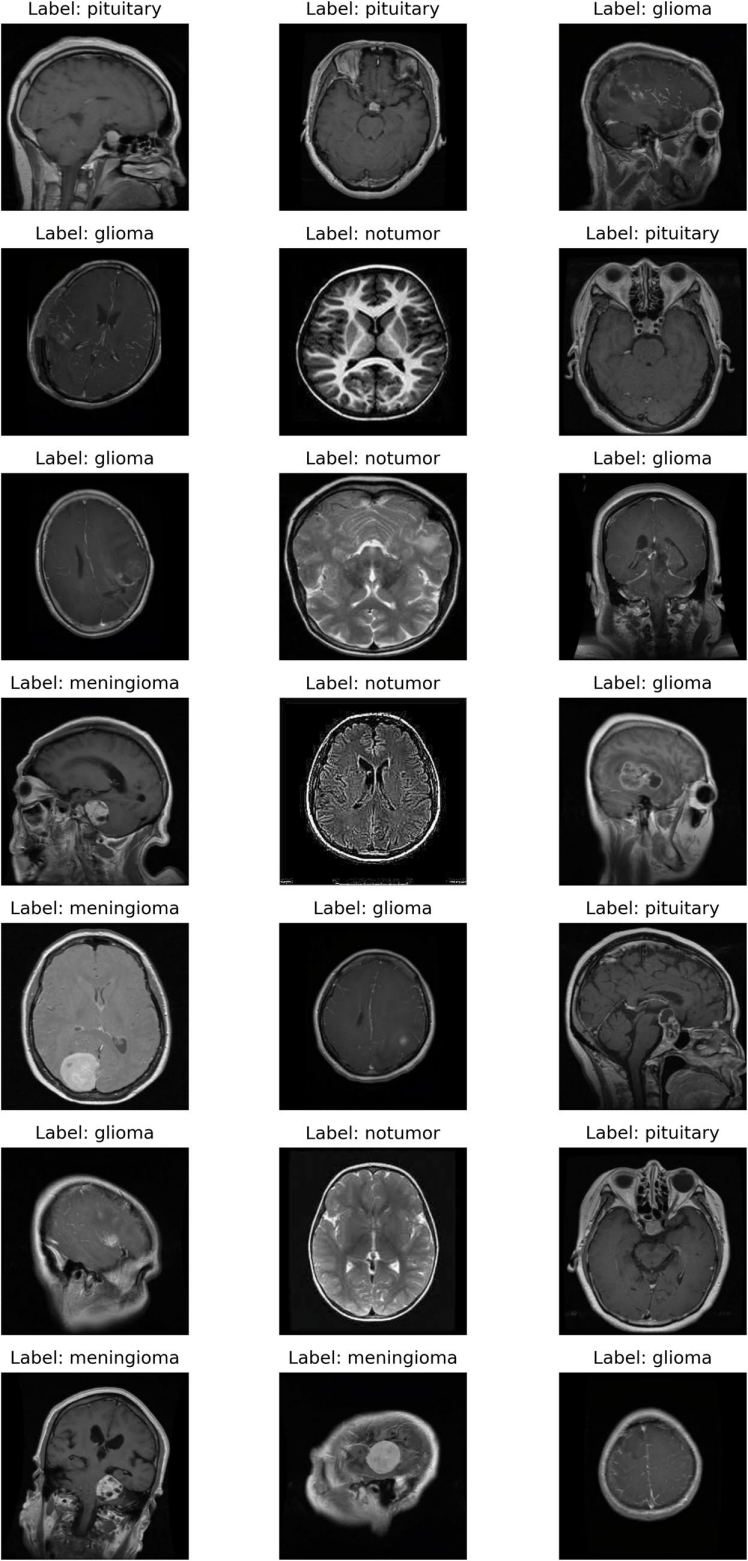
The labeling of the MRI images used in this study varied in both orientation (horizontal, sagittal) and type (T1, T2)

We compared five CNN architectures. Three of these models (ResNet-18, ResNet-34, and ResNet-50) were pretrained on the ImageNet dataset and subsequently fine-tuned for our specific classification task; fourth was ResNet-18 arichetaure without pretraining weights (Supplementary Fig. 1.) and the fifth was a custom CNN designed based upon a light and effiecent visual geometry group architecture (Supplementary Fig. 2.). We fine-tuned each model over 10 epochs on 5,712 training images, using AdamW (learning rate = 0.001, weight decay = 0.01) and a cosine-annealing learning rate scheduler. Performance was monitored on a dedicated validation set of 1,311 images, and the best-performing model checkpoint based on validation accuracy was retained for subsequent analysis. This approach leveraged the general feature representations learned from a large-scale dataset while allowing the networks to adapt to MRI-specific nuances. Figure 1 illustrates representative MRI scans labeled with each tumor type and the no tumor class.

### Model Development

**Fig. 2:**
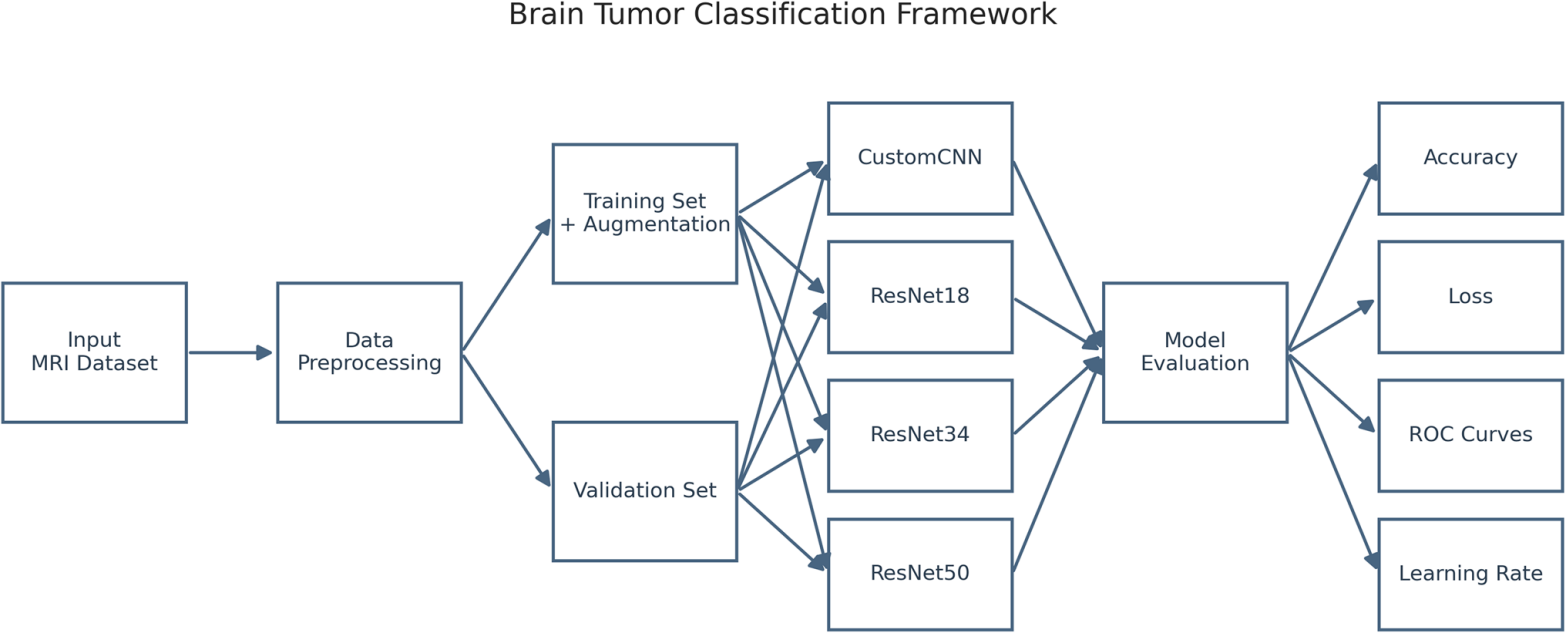
Overall framework for brain tumor classification. This diagram illustrates the end-to-end workflow for training and evaluating four convolutional neural networks-one custom-designed CNN and three pretrained ResNet variants (ResNet-18 scratch and pretrained, ResNet-34, and ResNet-50)-on a labeled brain MRI dataset. Raw images first undergo standardized preprocessing and are split into a training set (augmented to enhance variability) and a validation set. Each model is trained to differentiate between glioma, meningioma, pituitary tumor, and no tumor classes; subsequent performance is assessed via metrics including accuracy, loss, receiver operating characteristic (ROC) curves, and learning-rate progression

The model development was facilitated by Google's collaboration platform, which supported efficient data preprocessing, feature extraction, and model training phases. Figure 2 depicts the study framework pipeline.

### Hyperparameter Tuning and Training Settings

We tuned models with fivefold patient-wise cross-validation on the training set. To keep comparisons fair, we adopted a single configuration across models after pilot checks showed stable performance: AdamW (lr = 0.001, weight decay = 0.01) with CrossEntropyLoss, **a** cosine-annealing schedule (PyTorch *CosineAnnealingLR*, T_max = 10), batch size = 32, and 10 epochs. Augmentations (flip, rotation ± 15°, color jitter, small affine/scale) were applied to training data. For ResNet variants we replaced the final FC layer with a dropout-regularized classifier; pretrained ImageNet weights were used except for ResNet-18 (scratch), and the custom CNN was Kaiming-initialized. Cross-validated performance varied minimally across folds, so a broader search was unnecessary.

### Final Evaluation

Following this exploratory phase, we trained each model on the entire training set (*n* = 5,712) and evaluated it on the dedicated validation set (*n* = 1,311) for our final results. The single-split metrics reported in this manuscript closely matched those observed during cross-validation, indicating stable performance irrespective of the data partition.

### Model Performance Assessments

The model's performance was rigorously assessed through several statistical metrics: accuracy,, sensitivity, specificity, positive predicative value, negative predicative value, area under curve receiver operating characteristic (AUC-ROC) and the F1 score. These metrics were crucial for gauging the model's proficiency in correctly identifying tumor-positive cases, as well as its effectiveness in minimizing both false positives and false negatives. This comprehensive analysis provided critical insights into the model's diagnostic accuracy and potential clinical applicability.

### Experimental Setup and Evaluation

Detailed information about the experimental setup and training protocol is given in supplementary Table 1.

## Results

### Model Performance

**Fig. 3:**
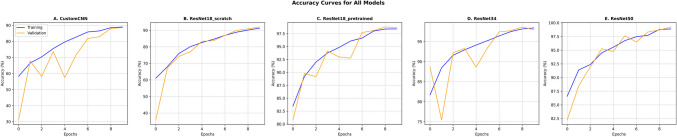
Training and validation accuracy curves for all CNN models across 10 epochs. Each panel tracks training (blue line) and validation (orange line) accuracies over 10 epochs for four CNN architectures: **A** a custom CNN, **B** ResNet-18 scratch (without pre trained weights), **C** ResNet-18 (**D**) ResNet-34, and (**E**) ResNet-50. While all models exhibit steady accuracy gains, the ResNet variants generally surpass the custom CNN and ResNet-18 scratch in final validation accuracy, with ResNet-18 pretrained and ResNet-50 closely exceeding 97%. These distinctions underscore the effect of leveraging pre-trained weights compared to training a network from scratch

The deep learning models demonstrated exceptional performance across all diagnostic categories. With ResNets models consistenly achivheing close to 100% accuracy (98%−99%), while the custom-CNN and ResNet-18 scratch(no pretrained weights) lagged behind in 87.03% and 91.99% accuracy respecitvely (Fig. 3). This high level of accuracy in both the training and validation phases indicates a robust model with excellent generalization capabilities. CustomCNN and ResNet18 (scratch) were significantly less accurate than the ResNet18 Pretrained [Δaccuracy − 8.53 percentage points (pp) and − 6.11 pp; both *p* < 0.01], while ResNet34 and ResNet50 were not significantly different (*p* = 0.216 and 0.541) (Table 1).

**Table 1:** Accuracy performance

Model	Accuracy (95% CI)	Δ Accuracy vs Resnet-18 pretrained (95% CI)	*P*-value (vs Resnet-18 pretrained)	AUC (95% CI)	*P*-value
ResNet18-pretrained (primary)	98.86% (98.25–99.39)	-	-	1.00 (0.99–1.00)	-
CustomCNN	90.31% (88.71–91.99)	− 8.53 pp (− 10.14– − 6.94)	** < 0.01**	0.98 (0.97–0.98)	0.73
ResNet18-scratch	92.75% (91.38–94.13)	− 6.11 pp (− 7.48– − 4.73)	** < 0.01**	0.98 (0.98–0.99)	0.81
ResNet34	98.17% (97.40–98.86)	− 0.69 pp (− 1.45– + 0.00)	0.21	0.999 (0.99–1.00)	0.9
ResNet50	98.55% (97.86–99.16)	− 0.30 pp (− 0.99– + 0.38)	0.54	1.000 (0.99–1.00)	0.94

### Diagnostic Ability

**Fig. 4:**
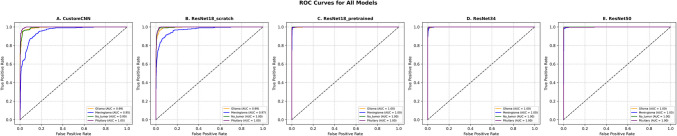
Multi-class ROC curves for all models. Each panel depicts the receiver operating characteristic (ROC) curves for the four brain tumor classes—glioma, meningioma, pituitary tumor—and the no tumor class, with the area under the curve (AUC) values annotated. All models demonstrate near-perfect discrimination (AUC ≥ 0.99 for most classes), underscoring their robust feature extraction and highly accurate classification performance

The AUC-ROC curves for each diagnostic category (glioma, meningioma, no tumor, and pituitary tumor) illustrate the model’s diagnostic prowess (Fig. 4.). All ResNet models achieved an area under the curve (AUC) of 1.00, indicating perfect discrimination between positive and negative cases for each category. With custom CNN and ResNet-18 scratch lagging behind but still with high results with AUC ranging between 0.94 and 1. Table 2 showcases the diagnostic performance metrics.

**Table 2:** Performance metrics for each model

**Model**	Diagnosis	Sensitivity	Specificity	PPV	NPV	AUC	**Accuracy**
Custom CNN; 4.7 M parameters							
	Glioma	0.79	0.99	0.95	0.94	0.98	0.94
	Meningioma	0.70	0.92	0.74	0.91	0.93	0.87
	Pituitary tumor	0.97	0.97	0.91	0.99	0.99	0.97
	No tumor	0.96	0.93	0.86	0.98	0.94	0.99
ResNet-18 scratch; 11.7 M parameters							
	Glioma	0.87	0.99	0.97	0.96	0.99	0.96
	Meningioma	0.82	0.95	0.85	0.94	0.96	0.92
	Pituitary tumor	0.98	0.96	0.92	0.99	0.99	0.97
	No tumor	0.96	0.93	0.86	0.98	0.94	0.99
ResNet-18 pretrained; 11.7 M parameters							
	Glioma	0.95	0.99	0.99	0.98	1	
	Meningioma	0.98	0.98	0.96	0.99	1	
	Pituitary tumor	0.99	0.99	0.98	0.99	1	
	No tumor	0.99	0.99	0.99	0.99	1	
ResNet-34; 21.2 M parameters							
	Glioma	0.99	0.99	0.99	0.99	1	0.99
	Meningioma	0.99	0.99	0.98	0.99	1	0.99
	Pituitary tumor	0.99	0.99	0.99	0.99	1	0.99
	No tumor	0.99	0.99	0.99	0.99	1	0.99
ResNet-50; 23.5 M parameters							
	Glioma	0.99	0.99	0.99	0.99	1	1
	Meningioma	0.98	0.98	0.96	0.99	1	0.99
	Pituitary tumor	0.99	0.99	0.98	0.99	1	1
	No tumor	1	1	1	0.99	1	1

### Error Analysis

**Fig. 5:**
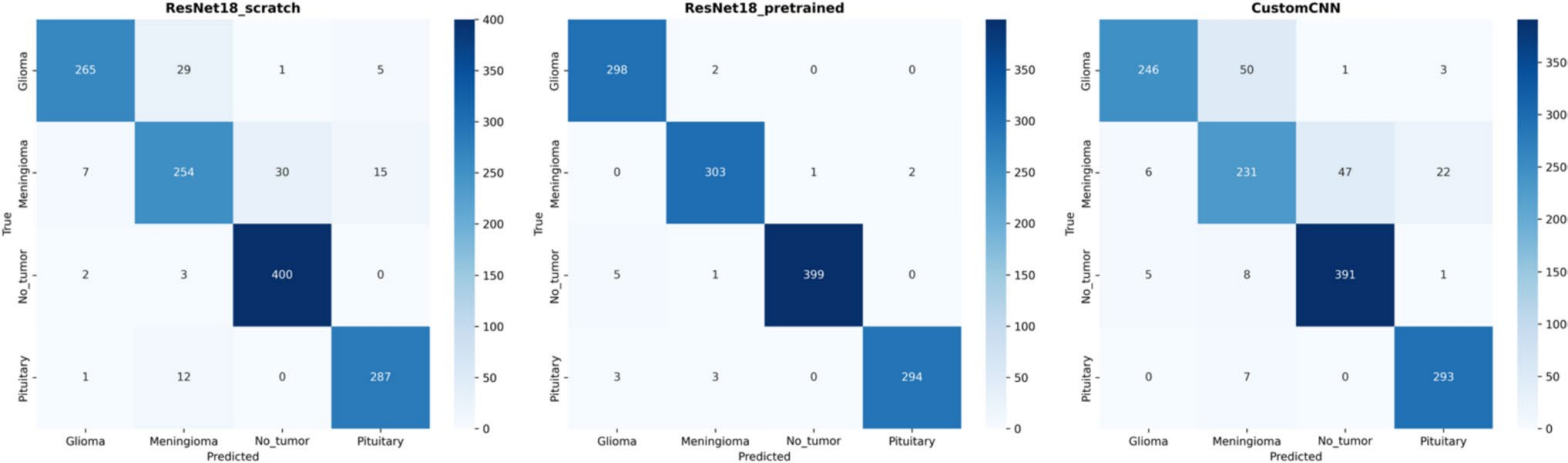
Confusion matrix of ResNet-18 scratch, ResNet-18 pretrained, and custom CNN. The confusion matrices compare the classification performance of three deep learning models ResNet-18 (trained from scratch), ResNet-18 (pretrained), and a custom CNN in detecting glioma, meningioma, no tumor, and pituitary tumor from MRI scans. Each matrix visualizes the true vs. predicted labels, with darker shades indicating a higher count of correctly classified cases. ResNet-18 (scratch) shows notable misclassification between glioma and meningioma. ResNet-18 (pretrained) exhibits superior accuracy, particularly for glioma and meningioma, with minimal misclassification. Custom CNN struggles more with distinguishing meningioma and no tumor cases compared to the pretrained model. These results highlight the effectiveness of transfer learning (pretrained ResNet-18) in improving classification accuracy for brain tumor detection

The confusion matrix of Resnet-18 scratch, pretrained weights, and custom-CNN provided a detailed view of the model's predictive accuracy across different classes (Fig. 5). The matrix showed predominant correct classifications with minimal confusion between classes, demonstrating the model's precise discriminatory capability between different tumor types and non-tumor types of MRI images. However for scratch ResNet-18 and custom-CNN there was moderarte confusion speifcally mistaken menignoma as glioma.

### Learning Rate Optimization

The learning rate schedule was strategically managed to optimize the training process (Supplementary Fig. 3.). Initially set high for broader exploration, the learning rate was gradually decreased to refine the model's convergence on optimal weights, facilitating robust learning without overfitting.

### Explainable AI Analysis

To enhance the interpretability of our deep learning models, we applied Explainable AI (XAI) techniques, including Grad-CAM and occlusion sensitivity maps, which provide transparency into the decision-making process of black-box CNNs [15, 18]. Grad-CAM produces heatmaps highlighting image regions most influential for predictions, while occlusion maps assess sensitivity by masking portions and observing confidence changes. Supplementary Fig. 4 displays these for representative true positive examples across glioma, meningioma, no tumor, and pituitary classes. In Grad-CAM heatmaps, warmer colors (red/yellow) focus on tumor-relevant areas, aligning with clinical features, whereas occlusion maps show blurred regions of high sensitivity. These visualizations build trust in model predictions, explain potential misclassifications (e.g., glioma-meningioma overlap), and support clinical adoption by clarifying AI focus.

### Loss Metrics

Notably, all five networks demonstrated a steady decrease in training loss, while validation loss remained relatively low and converged in tandem with training loss (Supplementary Fig. 5.). These trends suggest effective generalization rather than overfitting, corroborating the high accuracy and AUC scores observed in other metrics.

## Discussion

In this study, we systematically developed and evaluated multiple lightweight and efficient deep learning models specifically, ResNet-18 (both pretrained on ImageNet and trained from scratch), ResNet-34, ResNet-50, and a custom CNN for detecting glioma, meningioma, pituitary tumors, and non-tumorous cases using conventional MRI images. By conducting **a** side-by-side comparison**,** our work offers a granular view of how different levels of network depth and parameter counts affect diagnostic accuracy, especially when computational resources are limited. This focus on moderate-depth, high-efficiency architectures directly addresses the needs of smaller clinical centers where GPU power may be scarce yet accuracy demands remain high. Our research demonstrated that lightweight models, such as ResNet-18, can achieve exceptionally high performance through transfer learning equalling or in some cases surpassing the results of more parameter-heavy or complex architectures typically found in state-of-the-art (SOTA) models.

### Comparison with State-of-the-Art Approaches

Recent deep learning models for brain tumor classification have demonstrated remarkable accuracy, often exceeding 98%, by incorporating advanced architectural enhancements such as attention mechanisms, generative augmentation, and hybrid feature extraction techniques. However, these enhancements often come at the cost of increased computational complexity, making them less suitable for clinical environments with limited resources. For instance, SAlexNet [12] integrates a Hybrid Attention Mechanism (HAM) and residual layers to enhance feature extraction, achieving an accuracy of 99.69%. HAM helps SAlexNet capture both spatial and channel-wise dependencies, allowing it to enhance tumor localization and segmentation. Similarly, NeuroNet19 [10] builds upon VGG19 with an Inverted Pyramid Pooling Module (iPPM) to capture multi-scale features, attaining 99.3% accuracy. TumorGANet [9] employs ResNet-50 in conjunction with a GAN-based data augmentation strategy, reporting a classification accuracy of 99.53%. Additionally, Qureshi et al. [11] introduced an ultra-light CNN that combines deep learning with Gray-Level Co-occurrence Matrix (GLCM) texture analysis, reaching an accuracy of 99.23% while maintaining efficiency. While these models achieve high performance, their computational demands vary. SAlexNet and NeuroNet19 incorporate attention mechanisms and pooling modules, which may increase inference time, whereas TumorGANet relies on a GAN-based augmentation strategy that can add training complexity. However, Qureshi et al.'s ultra-light CNN emphasizes efficiency, demonstrating that smaller architectures can still achieve high accuracy. In contrast, our study focuses on "off-the-shelf" ResNet architectures, specifically ResNet-18, which balances simplicity with performance. Our approach demonstrates that strong data augmentation strategies, such as random affine transformations, color jittering, and rotation augmentation, can achieve SOTA performance without extensive architectural modifications, making it more practical for real-world deployment in smaller clinical settings. Moreover, when compared to traditional machine learning approaches such as artificial neural networks (ANNs) and random forests, our ResNet models demonstrates a clear advantage. For example, Hamd et al. [19] reported an AUC of 0.97 using ANN for pediatric brain tumor detection. In contrast, our ResNet-18 model achieved an AUC of 1.0 for glioma classification, underscoring the benefits of convolutional neural networks in learning spatial patterns and textures directly from MRI scans. The residual connections in ResNet further facilitate stable training by mitigating the vanishing gradient problem, enhancing feature extraction across deeper layers. Another key differentiator of our study is the systematic evaluation of multiple ResNet architectures, including ResNet-18, ResNet-34, and ResNet-50, to assess the impact of network depth and transfer learning on classification performance, providing a comparative perspective on computational efficiency versus accuracy. Unlike SAlexNet or NeuroNet19, which introduce additional attention and pooling mechanisms, our pipeline maintains a standardized architecture while leveraging strong data-level augmentations. This architectural simplicity enables easy integration into existing hospital frameworks, as ResNets are widely supported across various deep learning platforms used in clinical AI applications [20].

The ResNet model, a deep residual network architecture developed by He et al. [21], was pre-trained on the ImageNet dataset comprising over 14 million images. The use of ResNet allowed us to exploit its advanced feature extraction capabilities, resulting in more accurate and reliable detection of brain tumors compared to traditional ANN approaches. The higher AUC achieved by our CNN-based approach underscores the importance of using specialized deep-learning models like CNN with utilizing pre trained model such as ResNet-18 for medical image analysis. These models, pre-trained on extensive datasets, provide a robust framework for tackling complex medical imaging challenges, ultimately leading to improved diagnostic accuracy and better patient outcomes.

The reason we chose the CNN model for our model, was due to the CNN architecture is specifically designed to handle image data by leveraging layers that perform convolution operations to detect features such as edges, textures, and shapes within the images. These convolutional layers scan the MRI images with multiple filters to create feature maps that highlight important patterns. Pooling layers then reduce the dimensions of these feature maps, retaining critical information while minimizing computational load. Finally, fully connected layers combine these features to classify the images into their respective categories. This hierarchical feature extraction process allows CNNs to effectively capture the spatial and structural nuances of MRI scans, leading to accurate detection and differentiation of various brain tumor types. The ability of CNNs to automatically learn and extract relevant features from complex medical images makes them particularly suitable for medical image analysis, resulting in high diagnostic accuracy and reliability.

The application of AI in neuro-oncology has proven not only to enhance diagnostic accuracy but also to significantly improve efficiency. A notable competition involved a brain tumor diagnosis comparing the performances of human radiologists and an AI system developed by the Artificial Intelligence Research Centre for Neurological Disorders and Capital Medical University. In the competition the AI system, Biomind, achieved an 87% accuracy rate, diagnosing 195 out of 225 cases correctly within 15 min. In contrast, a team of 15 radiologists manually diagnosed 148 cases correctly, achieving a 66% accuracy rate over 30 min [22]. This demonstrates AI's potential to revolutionize diagnostic processes in neuro-oncology by delivering faster and more accurate results.

Based on the confusion matrix, we observed that 12 MRI images of glioma were incorrectly predicted as meningioma. Patel et al. reported similar diagnostic challenges, describing two cases where MRI initially suggested meningiomas, but surgical outcomes confirmed glioblastomas [23]. They attributed these diagnostic errors to gliomas exhibiting MRI characteristics typically associated with meningiomas, such as the dural tail sign, CSF cleft sign, and broad dural contact. These imaging mimicry patterns could account for the misdiagnoses observed, suggesting that even with the application of AI, misdiagnosis is possible due to the inherent similarities between diseases. This highlights a limitation within AI applications in neuro-oncology that must be addressed in future studies. Additionally, it's crucial to acknowledge that medical images often face issues like artifacts and resolution degradation, which can impact the accuracy of machine learning-based diagnostic methods.

The utilization of AI in neuro-oncology holds transformative potential by transitioning from exclusive reliance on radiologist expertise to standardized and efficient diagnostics accessible worldwide. This is facilitated by cloud-based platforms that provide virtual machines, making the primary requirement for utilizing the model just a basic computer. Wahl et al. highlight how expert systems like our deep learning model could enhance healthcare in resource-limited settings [24]. Such systems can assist physicians with diagnostics and treatment decisions, similar to practices in wealthier nations, and even substitute for human experts in areas where they are scarce [23]. This is particularly valuable in under-resourced communities. However, a significant challenge remains in these settings—the limited availability of MRI machines [25]. Addressing this issue requires a dual approach: integrating existing AI technologies to mitigate the absence of medical experts and increasing the accessibility of MRI technology in low-income countries.

Our study's strengths include the use of a pre-trained ResNet-18 CNN, which provided high diagnostic accuracy and AUC in classifying glioma, meningioma, pituitary tumors, and non-tumor MRI images. Utilizing publicly available Kaggle datasets ensures replicability and transparency. However, limitations exist, such as the use of 2D MRI slices, and potentially missing critical volumetric data from 3D images. The dataset's lack of clinical diversity may affect generalizability. Furthermore, the lack of longitudinal data and the necessity for additional clinical validation on larger databases underscore areas requiring future research.

## Conclusions

Our study evaluated multiple lightweight deep learning models for brain tumor classification, demonstrating that pretrained ResNet-18 achieves near-perfect accuracy (AUC 1.0, sensitivity/specificity > 97%) while remaining computationally efficient. Transfer learning significantly improved classification accuracy, reducing misclassification, particularly between gliomas and meningiomas. These findings support the deployment of AI-assisted diagnostics in resource-limited settings. Future work should incorporate multi-sequence MRI, 3D volumetric data, and real-world validation to enhance generalizability and clinical impact.

## Supplementary Information

Below is the link to the electronic supplementary material.

Supplementary Material 1 (DOCX 1.76 MB)

## Data Availability

The dataset used in this study is publicly available and can be accessed at [10.34740/kaggle/dsv/2645886].
