# Supplementary material for: Lightweight Transfer Learning Models for Multi-Class Brain Tumor Classification: Glioma, Meningioma, Pituitary Tumors, and No Tumor MRI Screening
Source: J Imaging Inform Med. 2025 Sep 19;39(3):2309–18. doi: 10.1007/s10278-025-01686-1 (PMC13230449; doi:10.1007/s10278-025-01686-1)
Supplement: Supplementary file 1 — Supplementary Material 1 (DOCX 1.76 MB) [file 10278_2025_1686_MOESM1_ESM.docx]

**Supplementary Tables**

**Supplementary table 1**

| Sr. No | Name | Value |
| --- | --- | --- |
| 1 | Computer model | MacBook Air (M1, 2020) |
| 2 | CPU | Apple M1, 16-core CPU |
| 3 | GPU | Apple M1 integrated GPU (16-core, model dependent) |
| 4 | Neural Engine | 16-core Apple Neural Engine |
| 5 | Memory (RAM) | 16 GB unified memory |
| 6 | Storage | 512 SSD |
| 7 | Operating system | macOS on Apple Silicon |
| 8 | Programming language | Python 3.12.x |
| 9 | Environment | venv + pip |
| 10 | Core libraries | numpy 2.x, pandas 2.2.x, scikit-learn 1.5.x, matplotlib 3.9.x |
| 11 | Deep-learning | PyTorch 2.x with MPS (Metal) backend for Apple Silicon |
| 13 | IDE / notebooks | JupyterLab |
| 14 | Train/test split | 80% training, 20% testing |

**Supplementary figures**

Figure 1. Original ResNet-18 Architecture


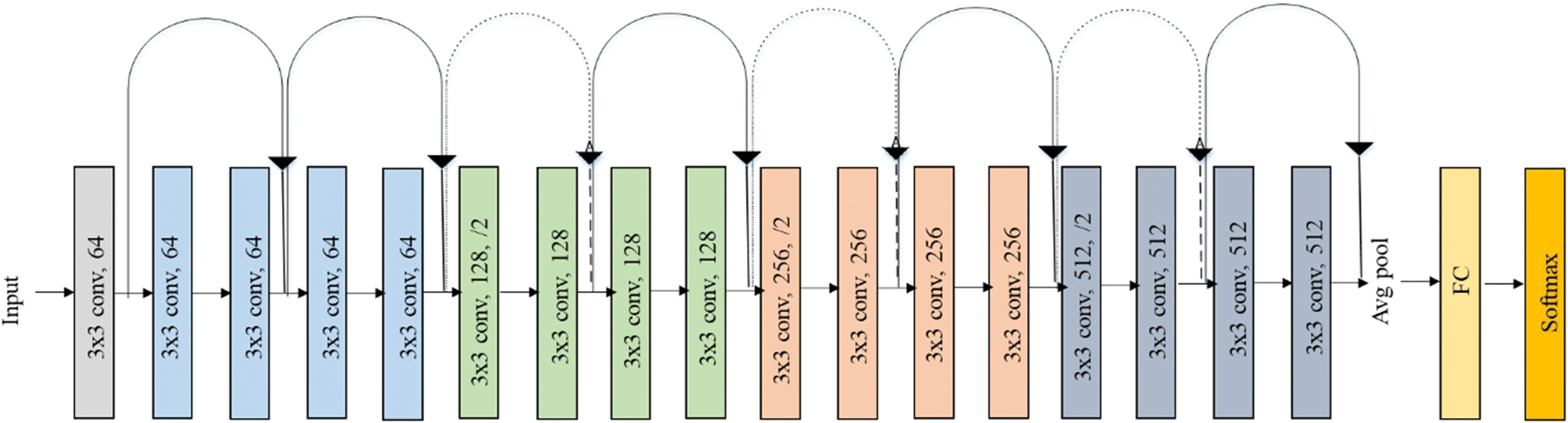


Figure 2. Schematic of the Custom Convolutional Neural Network Architecture for Brain Tumor Classification.


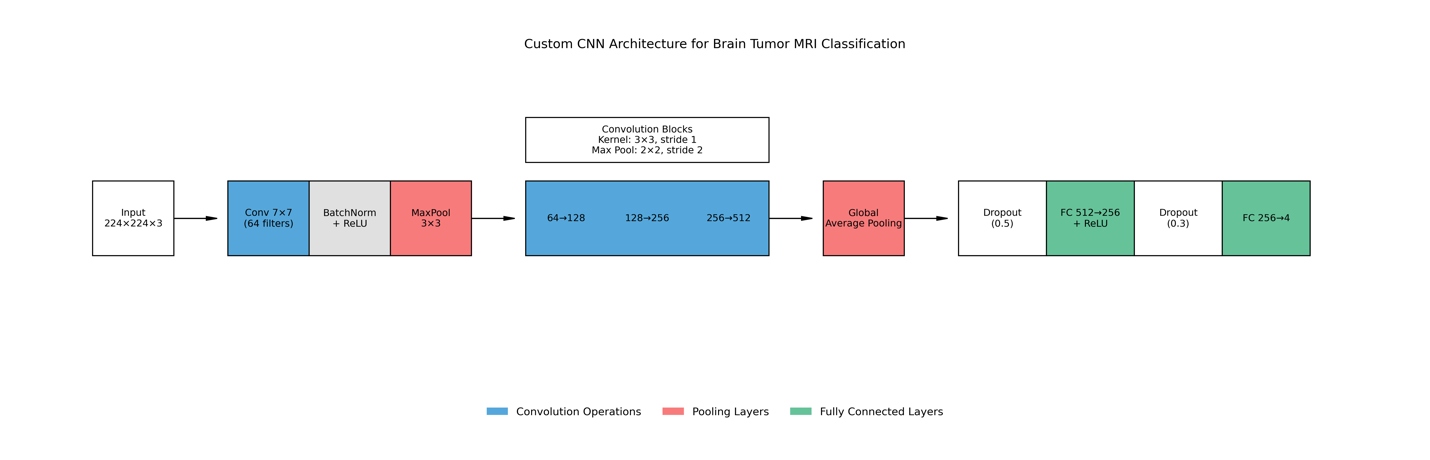


The model receives 224×224×3 input images, followed by a 7×7 convolution with 64 filters, batch normalization, ReLU activation, and a 3×3 max-pooling layer. Three subsequent convolutional blocks increase the filter depth (64→128, 128→256, 256→512) using 3×3 convolutions, batch normalization, and ReLU, each terminated by 2×2 max-pooling for progressive feature extraction. A global average pooling layer then condenses the final 512-channel feature maps to a 512-dimensional vector, which passes through fully connected layers with dropout of 0.5 and 0.3, respectively. The final 256→4 output layer produces logits corresponding to four classes-glioma, meningioma, pituitary tumor, and no tumor.

**Figure 3. Learning Rate Schedules for All Models.**


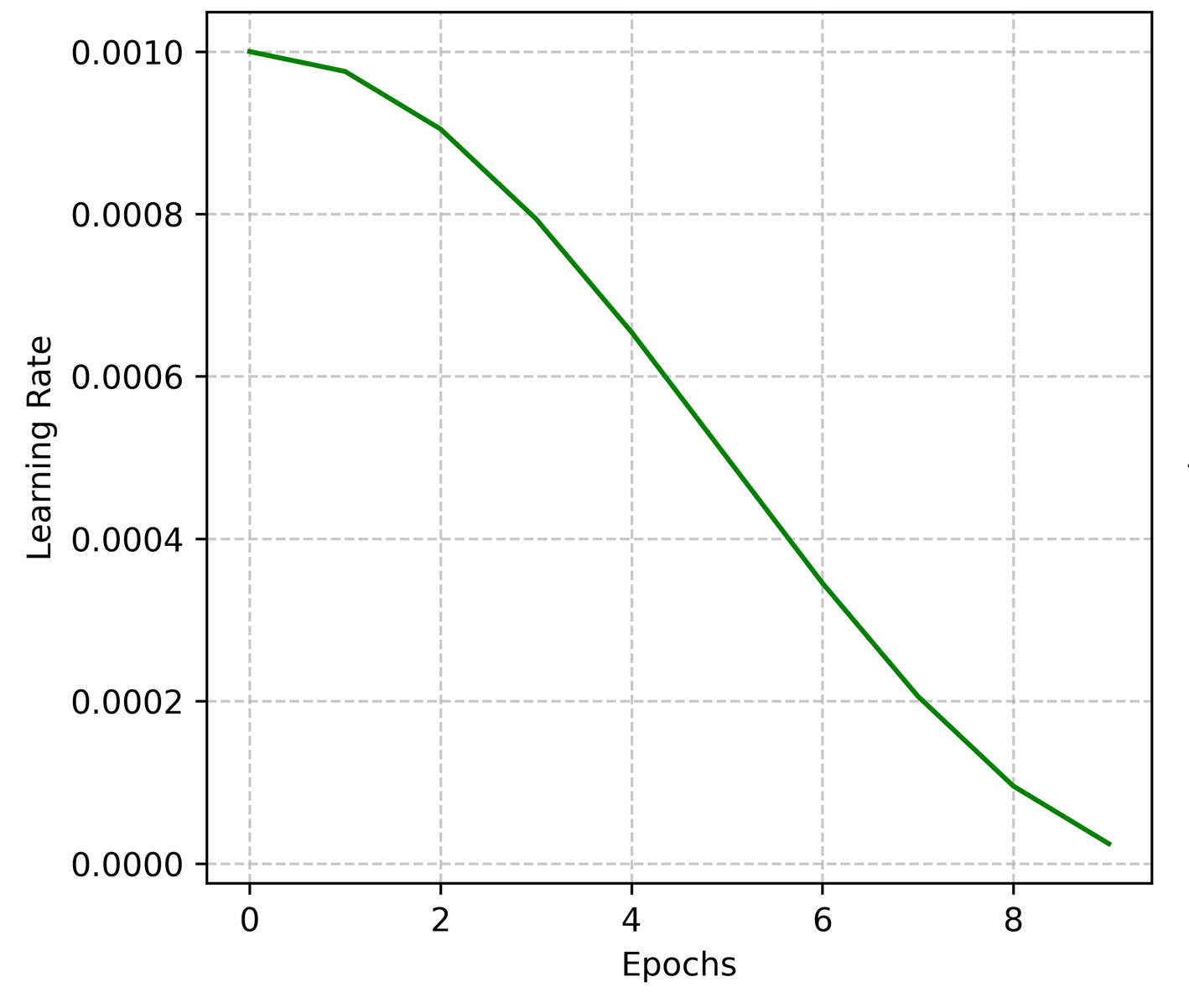


The plots illustrate the learning rate decay over 10 training epochs for each CNN. Driven by a cosine-annealing schedule beginning at 0.001, the rate gradually decreases, balancing initial exploration of the parameter space with later-stage fine-tuning. This approach promotes efficient convergence while mitigating the risk of overfitting.

**Figure 4 Grad-CAM and occlusion sensitivity for correctly classified test cases**
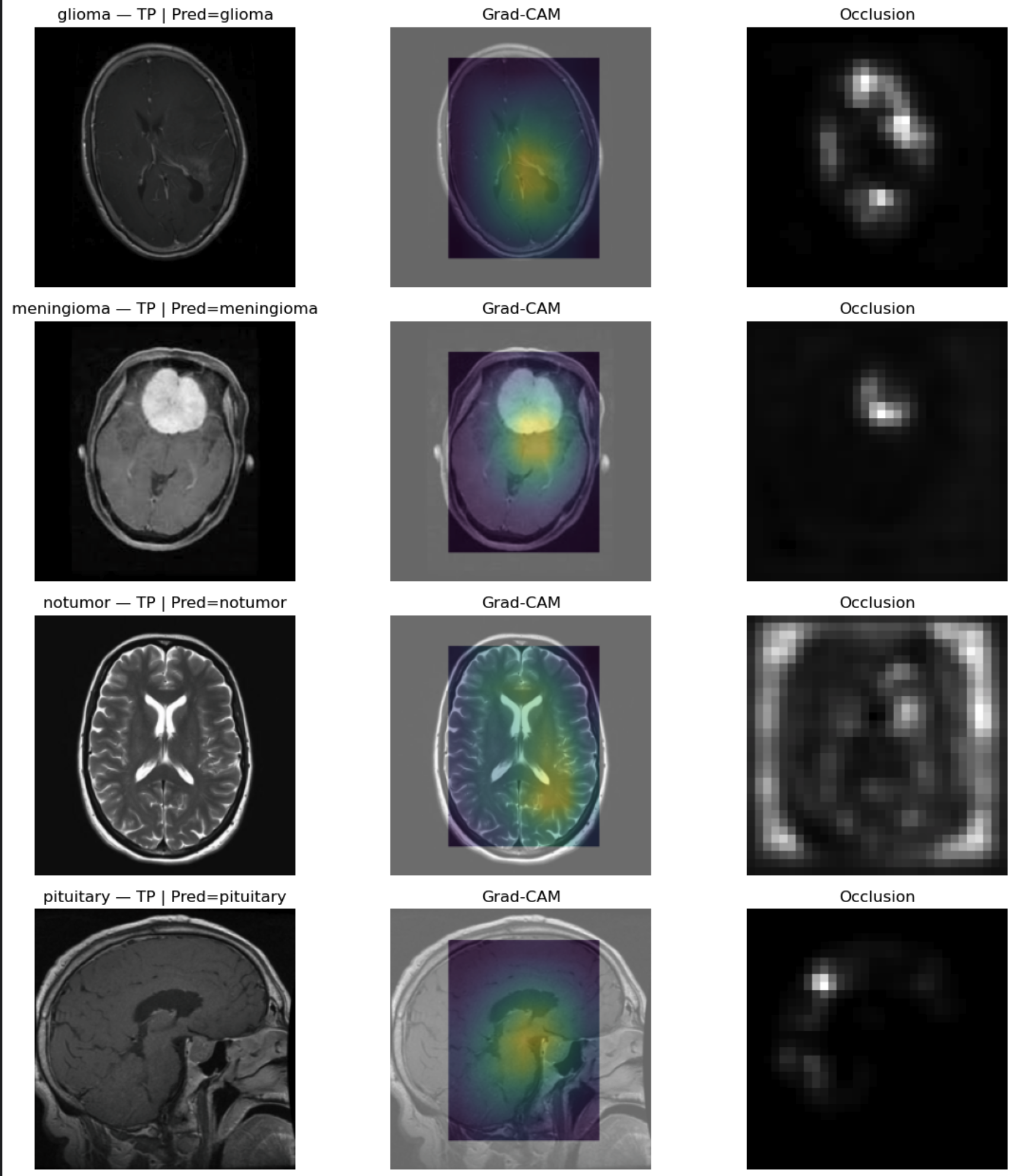


Left: input MRI slice. Middle: Grad-CAM heatmap overlaid on the slice (warmer colors indicate greater contribution to the predicted class). Right: occlusion sensitivity map using a sliding window (brighter regions cause the largest drop in confidence when masked). Examples are shown for glioma, meningioma, pituitary tumor, and no-tumor (notumor) classes. The highlighted regions align with disease-relevant anatomy, supporting that the primary model (ResNet-18 pretrained) attends to pathologic areas.

**Figure 5 Training and Validation Loss Curves for All Models.**


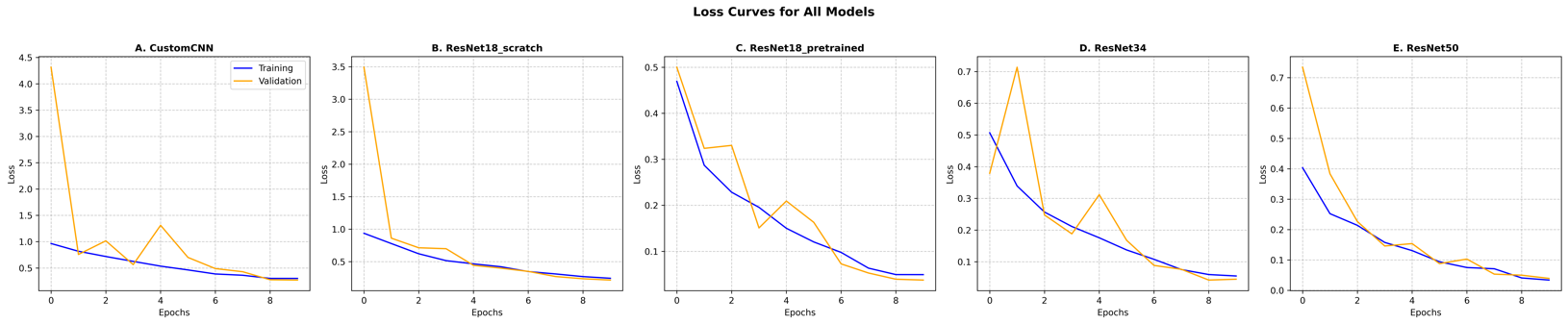
These curves show the progression of training (blue) and validation (orange) losses as each model learns from the dataset. The concurrent declines in training and validation loss, along with their close alignment, indicate effective learning and strong generalization performance across the CustomCNN, ResNet‑18, ResNet‑34, and ResNet‑50 architectures.
